# Physiological and Proteomic Responses of Contrasting Alfalfa (*Medicago sativa* L.) Varieties to High Temperature Stress

**DOI:** 10.3389/fpls.2021.753011

**Published:** 2021-12-09

**Authors:** Yingzhu Li, Xinrui Li, Jin Zhang, Daxu Li, Lijun Yan, Minghong You, Jianbo Zhang, Xiong Lei, Dan Chang, Xiaofei Ji, Jinchan An, Mingfeng Li, Shiqie Bai, Jiajun Yan

**Affiliations:** ^1^Institute of Herbaceous Plants, Sichuan Academy of Grassland Science, Chengdu, China; ^2^College of Grassland Science and Technology, Sichuan Agricultural University, Chengdu, China; ^3^Institute of Qinghai-Tibetan Plateau, Southwest Minzu University, Chengdu, China

**Keywords:** alfalfa, high temperature stress, physiological changes, isobaric tandem mass tag labeling (TMT), photosynthesis-responsive protein, metabolism-responsive protein, heat shock protein

## Abstract

High temperature (HT) is an important factor for limiting global plant distribution and agricultural production. As the global temperature continues to rise, it is essential to clarify the physiological and molecular mechanisms of alfalfa responding the high temperature, which will contribute to the improvement of heat resistance in leguminous crops. In this study, the physiological and proteomic responses of two alfalfa (*Medicago sativa* L.) varieties contrasting in heat tolerance, MS30 (heat-tolerant) and MS37 (heat-sensitive), were comparatively analyzed under the treatments of continuously rising temperatures for 42 days. The results showed that under the HT stress, the chlorophyll content and the chlorophyll fluorescence parameter (Fv/Fm) of alfalfa were significant reduced and some key photosynthesis-related proteins showed a down-regulated trend. Moreover, the content of Malondialdehyde (MDA) and the electrolyte leakage (EL) of alfalfa showed an upward trend, which indicates both alfalfa varieties were damaged under HT stress. However, because the antioxidation-reduction and osmotic adjustment ability of MS30 were significantly stronger than MS37, the damage degree of the photosynthetic system and membrane system of MS30 is significantly lower than that of MS37. On this basis, the global proteomics analysis was undertaken by tandem mass tags (TMT) technique, a total of 6,704 proteins were identified and quantified. Gene Ontology (GO) analysis and Kyoto Encyclopedia of Genes and Genomes (KEGG) analysis indicated that a series of key pathways including photosynthesis, metabolism, adjustment and repair were affected by HT stress. Through analyzing Venn diagrams of two alfalfa varieties, 160 and 213 differentially expressed proteins (DEPs) that had dynamic changes under HT stress were identified from MS30 and MS37, respectively. Among these DEPs, we screened out some key DEPs, such as ATP-dependent zinc metalloprotease FTSH protein, vitamin K epoxide reductase family protein, ClpB3, etc., which plays important functions in response to HT stress. In conclusion, the stronger heat-tolerance of MS30 was attributed to its higher adjustment and repair ability, which could cause the metabolic process of MS30 is more conducive to maintaining its survival and growth than MS37, especially at the later period of HT stress. This study provides a useful catalog of the *Medicago sativa* L. proteomes with the insight into its future genetic improvement of heat-resistance.

## Introduction

In recent years, the greenhouse effect has led to an upward trend in the global climate. Global warming predictions indicate that temperatures will increase another 2–6°C by the end of this century (Li et al., 2013; Sévellec and Drijfhout, 2018). Excessive temperature has a certain effect on plant growth and development, nutritional quality, yield and distribution range (Hinojosa et al., 2019; Kumar, 2020). Some studies have shown that the high temperature could greatly damage the potato yield and lead to the physiological defects of tuber (Rykaczewska, 2015). Therefore, it is very essential to study the impact of high temperatures (HT) on plant growth and development to maximize agricultural production and food security in the future (Li B. et al., 2018).

Heat stress caused by HT could cause damage to the plant growth. Such damage can be specifically divided into morphological, physiological and biochemical damage, which is interrelated and influence each other (Chen et al., 2016; Faiz et al., 2020). Under the condition of HT, the growth rate of plants is slow, the leaves are withered and yellow and the fruit of plants is often not full and abnormal (Krauss and Marschner, 1984; Guilioni et al., 2003; Shen et al., 2016). These phenotypic characteristics are closely related to the physiological changes of plants. Many types of environmental stresses both biotic and abiotic produce characteristic changes in physiology and metabolic processes of higher plants (Miteva et al., 2005). And under the HT stress, the metabolic process will change to maintain the homeostasis of plant. Some studies have indicated that the physiological and biochemical responses of plants to HT stress mainly consist of four systems that are membrane system, osmotic regulation system, antioxidant defense system and photosynthetic system (Ibrahim and Quick, 2001; Jia et al., 2016; Djanaguiraman et al., 2018; Pu et al., 2019). Biological membranes are highly ordered structures consisting of mosaics of lipids and protein, excessive temperatures can directly and effectively change the properties of these membranes, including their fluidity and permeability, which could lead to changes in the type and concentration of material exchange on both sides of the membrane (Zheng et al., 2011; Niu and Xiang, 2018). These changes act as a signal that can be transmitted to other systems and pathways. Osmotic regulating system is one of the main systems responding to these changes. The osmotic regulating system is mainly composed of some osmotic regulating substances, which are easy to dissolve and have small molecular weight, such as soluble proteins, proline, betaine and their derivatives (Guo, 2010; Liu et al., 2013). When cells need to be maintained or regulated, such substances can be rapidly generated and accumulated in large quantities, so as to improve the ability of water retention and acquisition, which is beneficial for maintaining the normal growth of plants (Wang et al., 2001). As a matter of fact, the main cause of damage to plants under stress is excessive reactive oxygen species (ROS), which is a class of substances that can react with macromolecules, such as lipids, DNA, RNA, and proteins, thereby destroying the structure and function of the cell (Kundu et al., 2020). Therefore, in order to prevent the excessive accumulation of ROS, plant cells have evolved a variety of antioxidant mechanisms to eliminate the toxicity caused by ROS, which is mainly divided into antioxidant enzymatic and non-enzymatic system (Navrot et al., 2007; Junmatong et al., 2015). Antioxidant enzymatic system mainly includes superoxide dismutase (SOD), peroxidase (POD), catalase (CAT) and non-enzymatic system is mainly some organic matter with small relative molecular mass, such as proline, ascorbic acid (ASA) and glutathione (GSH) (Ban et al., 2012; Chetty, 2017; Kolupaev et al., 2020). Compared with other systems, high temperature stress has more effect on photosynthetic system. Some studies have shown that under HT stress, thylakoid membrane was easy to be destroyed, the activity of photosynthesis-related enzymes was decreased or lost and the stomatal activity was drastically affected, which eventually leads to the decrease of net photosynthetic rate and the hindrance of the substances synthesis in plants (Schrader et al., 2004; Djanaguiraman et al., 2018; Sharma, 2020). It is worth noting that under the HT stress, the changes of phenotypic characteristics, physiological and biochemical processes in plants are regulated by complex genes networks (Wilkins et al., 2016; Li B. et al., 2018; Miguel et al., 2020). Proteomics has been a promising tool to reveal the dynamic response mechanism of proteins in plants under the biotic and abiotic stress (Hashiguchi et al., 2010; Sergeant and Renaut, 2010). Through comparative proteomic analysis, Mohammadi et al. (2014) found that proteins involved in cellular traffic, energy and metabolism, disease and defense, protein synthesis and signal transduction play an important role in the physiological and morphological responses of canola to HT stress. By studying the proteome of rice seeds, Liu et al. (2015) reported that high temperature could decrease the abundance of proteins involved in methionine metabolism, amino acid biosynthesis, energy metabolism, reserve degradation and protein folding and stress responses, which eventually inhibits seed germination. These studies will provide valuable information for understanding of HT-tolerant mechanisms in crops and pave the way for genetic engineering to improve crop resistance.

*Medicago sativa* L. is a high-quality forage due to its characteristics of high yield and excellent nutritional quality, which has the longest planting history and the largest planting area in the world (Chen et al., 2020). In China, alfalfa is one of the main herbages for animal husbandry and industrial feed production, it plays an important role in the industrialization of forage, the construction of artificial grassland and ecological environmental management (Li et al., 2015; Wang, 2020). In recent years, with the transformation and development of animal husbandry, the demand for forage with high yield and quality has increased year by year, which has led to an intense increase in the demand for alfalfa. In China, the planting area of alfalfa is mainly in the north, the introduced varieties in the south have the phenomena of decreasing yield, serious diseases and pests and quality deterioration, which is due to the high temperature climate affecting the normal growth of alfalfa (Lin et al., 2007; Xu et al., 2017; Anley, 2020). In fact, the damage of high temperature stress to alfalfa is one of the main factors limiting its popularization. And with global warming, high temperature weather in summer will be more frequent in China, which will seriously limit the production and utilization of alfalfa (Gao et al., 2019). Therefore, it is essential to study the heat tolerance mechanism of alfalfa and develop alfalfa cultivars that could cope better with stressful temperature events. In addition, with the development of liquid chromatography coupled to tandem mass spectrometry (LC-MS/MS), two popular quantitative proteomic approaches have been developed including label-free quantification (LFQ) and isobaric labeling strategy such as Tandem Mass Tags (TMT) and isobaric Tags for Relative and Absolute Quantitation (iTRAQ) (Ulsen et al., 2009; Nogueira et al., 2012; Kawashima et al., 2019). Compared with conventional 2-DE technique, Tandem Mass Tags (TMT) based quantitative proteomics could more efficiently and accurately analyze more numerous proteins (Pagel et al., 2015). However, the current research studies on alfalfa stress resistance mainly focus on cold resistance, drought resistance and salt-alkaline resistance (Zhang et al., 2017, 2018; Liu et al., 2020). And reports on heat resistance of alfalfa are relatively scattered, and quantitative proteomics studies on heat-stressed alfalfa leaves based on TMT analysis have not been reported. Based on the above, in the present study, TMT is performed to identify the global changes in protein expression under HT treatment in MS30 and MS37, which is screened according to our previous experimental results (Supplementary Table 1) and respectively represent HT-tolerant and HT-sensitive alfalfa varieties. Furthermore, we comprehensively compare the physiology and proteomic changes of these two alfalfa varieties under continuously increasing HT stress, characterize the HT-responsive proteins, and analyzed the functions of these differentially expressed proteins (DEPs) in order to clarify differential physiological and proteomic responses of two different heat resistant alfalfa varieties to HT stress. The results of this study could enhance the current understanding of the protein changes underlying HT stress-related cellular and physiological responses between HT-resistant and HT-sensitive alfalfa varieties, and provide basic data and technical theoretical support for the genetic improvement of high-quality and heat-resistant alfalfa varieties.

## Materials and Methods

### Plant Growth Conditions and Stress Treatments

Two alfalfa varieties contrasting in heat tolerance, MS30 and MS37, were used for this experiment, and their seeds were provided by Sichuan Academy of Grassland Sciences. HT-tolerant MS30 and HT-sensitive MS37 were screened according to our previous experimental results (Supplementary Table 1), and the specific experimental methods and the selection of experimental indicators are based on Zhang et al. (2010), Liu et al. (2014), and Li (2015). The seeds of two alfalfa varieties were surface sterilized with 10% sodium hypochlorite solution and repeated washes with sterilized distilled water. The washed seeds were sown in a nutritional bowl (20 cm in diameter and 18 cm in height) filled with natural soil 0.45 kg (30%), nutrient soil 0.6 kg (40%), perlite 0.225 kg (15%) and vermiculite 0.225 kg (15%). Natural soil, nutrient soil, perlite and vermiculite were disinfected by the corresponding methods to reduce the influence of bacteria and worm eggs on alfalfa growth. Seeds of each alfalfa variety were sown in three pots as three biological repeats. The nutritional bowls were placed in a growth chamber with controlled conditions (day/night cycle: 16/8 h, 20/15°C, 60 ± 5% relative humidity, 400 μE⋅m^–2^⋅s^–1^ PPFD). After 2 months of growth, all alfalfa varieties grow into adult-plants. Select 10–15 healthy plants with consistent growth to remain in each nutritional bowl. All materials are transferred to artificial climate boxes (day/night cycle: 16/8 h, 60 ± 5% relative humidity, 400 μE⋅m^–2^⋅s^–1^ PPFD) for high temperature stress treatment. According to the results of a prior experiment and the relevant report, the treatment temperature was set to 20, 25, 30, 35, 40, and 43°C, which is a gradient upward trend. Among them, alfalfa growing at 20°C were used as the control (non-HT stress). All alfalfa varieties were treated at each temperature gradient for 7 days and the leaves was collected for the determination of physiological indicators. Moreover, the physiological conditions of the plants and the humidity of the climate box were observed at any time. Water the plants properly once a day to ensure the water demand of the plants. Based on the results of the above experiment, some key physiological responses demonstrated that both alfalfa varieties showed the most significant variation at 20, 35, and 43°C. Therefore, leaf samples collected from plants after exposure to 20, 35, and 43°C for 7 days, respectively, were harvested for further TMT quantitative proteomic analysis.

### Physiological Measurements

The indicators of membrane system, osmotic regulation system, antioxidant defense system and photosynthetic system were measured in each treatment group. About the photosynthetic system, the chlorophyll content of the leaves was measured by a chlorophyll meter (SPAD-502; Konica Minolta Sensing, Inc., Osaka, Japan). The measurements were made on the axial surface of 10 different green leaves taken from each nutritional bowl (Sharma et al., 2015). Chlorophyll fluorescence was measured by a Chlorophyll Fluorescence Imager (CF Imager; Technologica, Inc., United Kingdom). The plants were dark adapted for 30 min, then the five measured leaves were illuminated with a 2 s saturating light flash at 3,000 μmol photons m^–2^ s^–1^ for CF Imager. The fluorescence data were analyzed and Fv/Fm ratio was calculated using the CF Imager software (version 1.10) (Sharma et al., 2012). About the membrane system, Malondialdehyde (MDA) content was measured by the thiobarbituric acid (TBA) reaction using the method described by Chołuj et al. (2014). Briefly, the leaves (0.1 g) were grinded, homogenized in 10 mL 10% trichloroacetic acid (TCA), and centrifuged at 1,500 × *g* for 10 min. Then 2 ml supernatant was isovolumetric mixed with 0.6% TBA and boiled at 100°C for 10 min. The absorbance values at 532 nm and 600 nm were used to calculate the MDA content. Each sample has three biological repeats. Electrolyte leakage (EL) was detected by using a conductivity meter (YSI Model 32, Yellow Spring, OH, United States) and calculated as the percentage of initial conductivity (Cinitial) and maximum conductance (Cmax) (Blum and Ebercon, 1981). About the osmotic regulation system, the soluble protein content was measured using bovine serum albumin as described by Bradford (1976). The soluble sugar content was measured according to Buysse and Merckx (1993). The leaves (0.1 g) were grinded and added into 10 mL 80% ethanol and then extracted in boiled water (100°C) for 15 min. After centrifugation at 3600 g_n_ for 10 min, supernatants were collected for further analysis. The reaction mixture contained 1 mL of supernatant, 1 mL of 18% phenol solution, and 5 mL concentrated sulfuric acid. The mixture was shaken, and absorbance was read at 490 nm using a spectrophotometer (Spectronic Instruments). The free proline content was determined using the acid ninhydrin method (Bates et al., 1973). The leaves (0.1 g) were grinded and immerged in 5 mL 3% aqueous sulfosalicylic acid and then heated in a water bath (100°C) for20 min. After centrifugation at 12,000 g_n_, the supernatant was used for the estimation of the proline concentration. The reaction mixture consisted of 1 mL of supernatant, 1.5 mL of 2.5% acid ninhydrin, and 1 mL of glacial acetic acid, which was heated at 100°C for 40 min. The reaction mixture was immediately cooled down in an ice bath, and extracted with 2.5 mL of toluene then absorbance was read at 520 nm.

About the antioxidant defense system, the extraction procedure of the enzymes was carried out at 4°C (Chao et al., 2010). The leaves were homogenized (1:10 w/v) in an ice cold mortar using 50 mM sodium phosphate buffer pH 7.0 containing 0.5 M NaCl, 1 mM EDTA and 1 mM sodium ascorbate. After centrifugation (20,000 × *g*, 20 min) the supernatant was used for the determination of SOD, POD, CAT, APX and GSH-Px activities. The SOD activity was measured at 560 nm according to Giannopolitis and Ries (1977). An amount of 50 μl of crude extract was added in a 1.5-ml reaction solution containing 195 mM methionine, 3 μM EDTA, 1.125 mM NBT, 60 μM riboflavin, and 50 mM PBS buffer (pH 7.8). The reduction of absorbance was recorded at 560 nm with a spectrophotometer. The POD activity was assayed at 470 nm as described by Chance and Maehly (Machly and Chance, 1955). The assay mixture contained 50 mM sodium acetate buffer pH 5.6, 5.4 mM guaiacol, 15 mM H_2_O_2_ and enzyme extract. The increase in absorbance due to the oxidation of guaiacol to tetraguaiacol was monitored at 470 nm. The activities of CAT and APX were assayed by the method of Nakano and Asada (1981). Briefly, the assay mixture contained 50 mM sodium phosphate buffer pH 7.0, 15 mM H_2_O_2_ and enzyme extract. Decomposition of H_2_O_2_ was measured at 240 nm. The assay mixture consisted of 50 mM sodium phosphate buffer pH 7.0 containing 1 mM EDTA, 0.25 mM sodium ascorbate, 25 μM H_2_O_2_ and enzyme extract. Addition of H_2_O_2_ started the reaction. Rates were corrected for the non-enzymatic oxidation of ascorbate by the inclusion of reaction mixture without enzyme extract. The GSH-Px activity was assayed at 470 nm as described by Wheeler et al. (1990). The mixture contained 50 mM potassium phosphate buffer pH 7.0, 2 mM EDTA, 150 mM GSH, 4.2 mM NADPH, 0.5 unit of glutatione reductase and 2.2 mM *t*-butyl peroxide, which started the reaction. The absorbance of the reacted solution at 340 nm was determined with a spectrophotometer. The levels of the above physiological parameters were all calculated based on fresh weight.

### Protein Extraction

The sample was grinded by liquid nitrogen into cell powder and then transferred to a 5-mL centrifuge tube. After that, four volumes of lysis buffer (8 M urea, 1% Triton-100, 10 mM dithiothreitol, and 1% Protease Inhibitor Cocktail) was added to the cell powder, followed by sonication three times on ice using a high intensity ultrasonic processor (Scientz) (Note: For PTM experiments, inhibitors were also added to the lysis buffer, e.g., 3 μM TSA and 50 mM NAM for acetylation). The remaining debris was removed by centrifugation at 20,000 *g* at 4°C for 10 min. Finally, the protein was precipitated with cold 20% TCA for 2 h at −20°C. After centrifugation at 12,000 *g* 4°C for 10 min, the supernatant was discarded. The remaining precipitate was washed with cold acetone for three times. The protein was redissolved in 8 M urea and the protein concentration was determined with BCA kit according to the manufacturer’s instructions.

### Trypsin Digestion and Tandem Mass Tags Labeling

For digestion, the protein solution was reduced with 5 mM dithiothreitol for 30 min at 56°C and alkylated with 11 mM iodoacetamide for 15 min at room temperature in darkness. The protein sample was then diluted by adding 100 mM TEAB to urea concentration less than 2 M. Finally, trypsin was added at 1:50 trypsin-to-protein mass ratio for the first digestion overnight and 1:100 trypsin-to-protein mass ratio for a second 4 h-digestion. After trypsin digestion, peptide was desalted by Strata × C18 SPE column (Phenomenex) and vacuum-dried. Peptide was reconstituted in 0.5 M TEAB and processed according to the manufacturer’s protocol for TMT kit. Briefly, one unit of TMT reagent were thawed and reconstituted in acetonitrile. The peptide mixtures were then incubated for 2 h at room temperature and pooled, desalted and dried by vacuum centrifugation.

### Liquid Chromatography-Tandem Mass Spectrometry Analysis and Database Search

The tryptic peptides were dissolved in 0.1% formic acid (solvent A), directly loaded onto a home-made reversed-phase analytical column (15-cm length, 75 μm i.d.). The gradient was comprised of an increase from 6 to 23% solvent B (0.1% formic acid in 98% acetonitrile) over 26 min, 23 to 35% in 8 min and climbing to 80% in 3 min then holding at 80% for the last 3 min, all at a constant flow rate of 400 nL/min on an EASY-nLC 1000 UPLC system.

The peptides were subjected to NSI source followed by tandem mass spectrometry (MS/MS) in Q ExactiveTM Plus (Thermo) coupled online to the UPLC. The electrospray voltage applied was 2.0 kV. The m/z scan range was 350 to 1800 for full scan, and intact peptides were detected in the Orbitrap at a resolution of 70,000. Peptides were then selected for MS/MS using NCE setting as 28 and the fragments were detected in the Orbitrap at a resolution of 17,500. A data-dependent procedure that alternated between one MS scan followed by 20 MS/MS scans with 15.0 s dynamic exclusion. Automatic gain control (AGC) was set at 5E4. Fixed first mass was set as 100 m/z.

The resulting MS/MS data were processed using Maxquant search engine (v.1.5.2.8). Tandem mass spectra were searched against human uniprot database concatenated with reverse decoy database. Trypsin/P was specified as cleavage enzyme allowing up to 4 missing cleavages. The mass tolerance for precursor ions was set as 20 ppm in First search and 5 ppm in Main search, and the mass tolerance for fragment ions was set as 0.02 Da. Carbamidomethyl on Cys was specified as fixed modification and acetylation modification and oxidation on Met were specified as variable modifications. FDR was adjusted to <1% and minimum score for modified peptides was set >40. The mass spectrometry proteomics data have been deposited to the ProteomeXchange Consortium via the PRIDE partner repository with the dataset identifier PXD025642.

### Parallel Reaction Monitoring Validations

To verify the protein expression levels obtained by TMT analysis, 14 DEPs (unique peptides ≥ 2, fold change > 1.2) were randomly chosen based on the TMT results and further quantified by the parallel reaction monitoring (PRM) assay according to Xu’s method (Xu et al., 2018) at Jingjie PTM-Biolab Co., Ltd. (Hangzhou, China). Briefly, peptides were prepared as above described for TMT assay. These obtained peptide mixtures were subjected to NSI source followed by tandem mass spectrometry (MS/MS) in Q ExactiveTM Plus (Thermo) coupled online to the UPLC. A full MS was performed in the Orbitrap at a resolution of 70,000 (AGC target at 3E6; the maximum injection time at 50 ms and the m/z range was 350–1200), followed by 20 MS/MS scans on the Orbitrap at a resolution of 17,500 (AGC target was 1E5, and the maximum injection time was 100 ms). Mass window for precursor ion selection was 1.6 m/z. The isolation window for MS/MS was set at 2.0 m/z. The NCE was 27% with HCD. The FDR was set to 0.01 for the proteins and peptides. The resulting MS data were processed using Skyline (v.3.6) program as described before (Kim et al., 2016).

### Statistical Analysis

All physiological data analyses were performed using the SPSS Statistical Software (version 19.0; SPSS Institute Ltd., United States), and the significance of differences were tested using Fisher’s protected least significant difference test (LSD) with a *P*-value ≤ 0.05 set as statistically significant.

## Results

### Effect of High Temperature Stress on Physiological Level of Alfalfa

#### Effect of High Temperature Stress on Photosynthetic System

**FIGURE 1:**
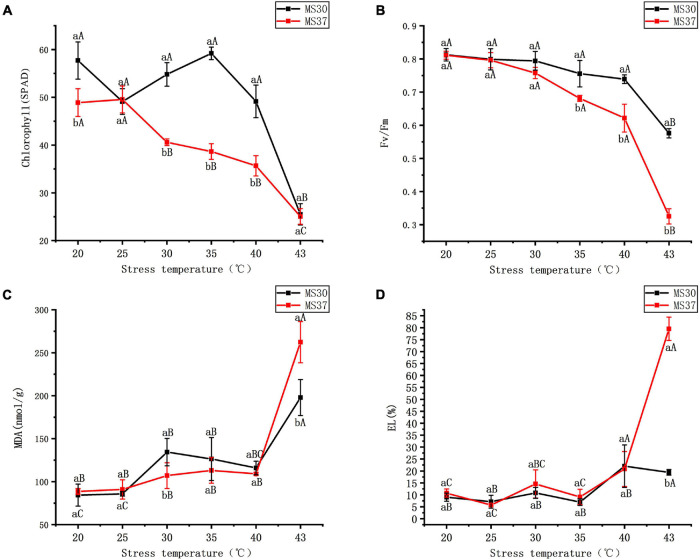
The changes of photosynthetic system and membrane system in MS30 and MS37 under HT stress. **(A)** Total chlorophyll content. **(B)** The chlorophyll fluorescence parameter (Fv/Fm). **(C)** MDA content. **(D)** Electrolyte leakage. Different capital letters above line graphs indicate significant difference among various temperature treatments within the same alfalfa varieties (*p* < 0.05), different little letters above line graphs indicate significant difference among two alfalfa varieties under the same temperature (*p* < 0.05). Data are means ± SE from measurements of three replicate experiments.

Under the HT stress, the variation trend of chlorophyll content in the two alfalfa varieties was different (Figure 1A and Supplementary Table 2). The heat resistant alfalfa variety (MS30) had the highest chlorophyll content at 35°C, and then significant decreased with the increasing of temperature. In the condition of 43°C, the chlorophyll content of MS30 is significantly lower than that of the CK by 32.19 SPAD (*p* < 0.05). But for MS37, the chlorophyll content was no significant change under 20 and 25°C, and with the increase of temperature, the chlorophyll content decreased obviously. At the condition of 43°C, the chlorophyll content was only 25.06 SPAD, which is similar to MS30. During the period of HT stress, the chlorophyll fluorescence parameter Fv/Fm in the two alfalfa varieties showed a gradual declining trend with the increased temperature (Figure 1B and Supplementary Table 2). As the temperature increased from 20 to 43°C, the decreased range of Fv/Fm in MS37 is obviously higher than that of MS30. The Fv/Fm value of MS37 was only 0.33 under the 43°C, which was significantly lower than that of MS30 under this condition (*p* < 0.05).

#### Effect of High Temperature Stress on Membrane System

Under the condition of the HT stress, the membrane system of two alfalfa varieties were all effected to certain degrees (Figures 1C,D and Supplementary Table 2). As the temperature gradually increases, the content of Malondialdehyde (MDA) in the two alfalfa varieties were significantly higher than that in control (*p* < 0.05). Under the condition of 43°C, the content of MDA in the two alfalfa varieties were highest, which of MDA in MS30 was significantly lower than that in MS37 (*p* < 0.05). And with the increase of temperature, the electrolyte leakage (EL) of the two alfalfa varieties showed an upward trend. But it is worth noting that the varied degree of EL in MS37 were significantly greater than those of MS30 under the HT stress. The EL of MS30 peaked at the condition of 43°C, which is significantly higher than MS37 (*p* < 0.05).

#### Effect of High Temperature Stress on Osmotic Regulation System

**FIGURE 2:**
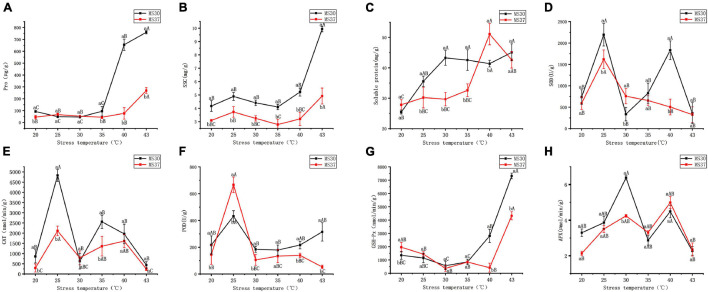
The changes of Osmotic Regulation System and Antioxidant Defense System in MS30 and MS37 under HT stress. **(A)** Pro content. **(B)** Soluble sugar content (SSC). **(C)** Soluble protein content. **(D)** The activity of superoxide dismutase (SOD). **(E)** The activity of catalase (CAT). **(F)** The activity of peroxidase (POD). **(G)** The activity of glutathione peroxidase (GSH-Px). **(H)** The activity of ascorbate peroxidase (APX). Different capital letters above line graphs indicate significant difference among various temperature treatments within the same alfalfa varieties (*p* < 0.05), different little letters above line graphs indicate significant difference among two alfalfa varieties under the same temperature (*p* < 0.05). Data are means ± SE from measurements of three replicate experiments.

With the increased intensity of high temperature stress, the osmotic regulation system in both alfalfa varieties showed an apparent change (Figures 2A–C and Supplementary Table 2). The two alfalfa varieties content of proline peaked at the condition of 43°C. And compared with the proline content under the control, the proline content of MS30 and MS37 increased by 667.49 and 223.68 μg/g, respectively. It is noteworthy that the proline content of MS30 had increased to 754.82 μg/g at the 43°C, which was significantly higher than this of MS37 by 577.64 μg/g (*p* < 0.05). This result indicates that under the HT stress, the response of osmotic adjustment substances in heat-resistant alfalfa variety, MS30, are better than those of heat sensitive alfalfa variety, MS37. The changes of soluble sugar content (SSC) in the two alfalfa varieties under HT stress also prove this result. The SSC in MS30 peaked at the condition of 43°C, which is significantly greater than this of MS37 (*p* < 0.05). Under HT stress, the content of soluble protein in the two alfalfa varieties generally showed an increasing trend, and the soluble protein content of MS37 Peaked at the condition of 40°C. However, at the 43°C, the soluble protein content of MS30 was similar to this of MS37, which is 45.07 and 42.62 mg/g, respectively.

#### Effect of High Temperature Stress on Antioxidant Defense System

According to the results of Figure 2, the superoxide dismutase (SOD) and catalase (CAT) activities of MS30 were higher than that of MS37 in all the treatments except for 30°C (Figures 2D,E and Supplementary Table 2). The maximum and minimum values of SOD and CAT activities in the two alfalfa varieties were both detected at 25 and 43°C. The activities of peroxidase (POD) in the two alfalfa varieties peaked at 25°C, and the POD activity of MS37 was significantly higher than that of MS30 (*p* < 0.05) (Figure 2F). But when the temperature gradually increases to 43°C, the POD activity of MS30 was 1515 U/g, which was significantly greater than that of MS37 (*p* < 0.05). In the early stage of HT stress, the glutathione peroxidase (GSH-Px) activities of the two alfalfa varieties all declined (Figure 2G). But in the later stage of HT stress, the GSH-Px activities of the two alfalfa varieties both showed an upward trend, and the GSH-Px activities in MS30 were significantly greater than that of MS37 at 43°C (*p* < 0.05). Under the HT stress, the ascorbate peroxidase (APX) variation trends of the two alfalfa varieties were consistent (Figure 2H). And the APX activities of MS30 and MS37 are both decreased at 43°C, which was respectively 2.27 and 2.35 μmol/min/g.

### Effect of High Temperature Stress on Proteome of Alfalfa

**FIGURE 3:**
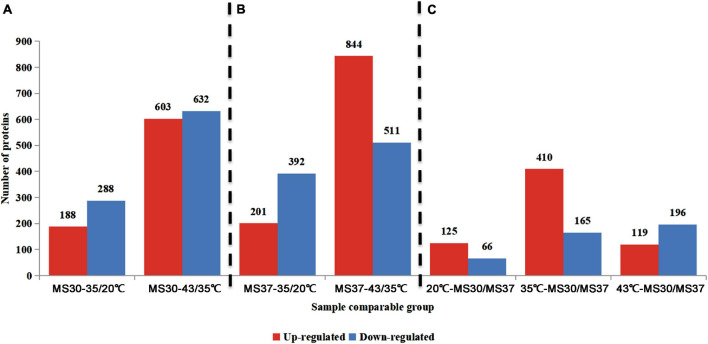
The number of DEPs in different comparison groups. **(A)** The comparison groups of MS30. MS30-35/20°C stands for the proteome of MS30 at 35°C compared with that of MS30 at 20°C. MS30-43/35°C stands for the proteome of MS30 at 43°C compared with that of MS30 at 35°C. **(B)** The comparison groups of MS37. MS37-35/20°C stands for the proteome of MS37 at 35°C compared with that of MS37 at 20°C. MS37-43/35°C stands for the proteome of MS37 at 43°C compared with that of MS37 at 35°C. **(C)** The comparison groups of MS30 and MS37 under the same temperature. The data on the columns indicate the number of up- or down-regulated expressed DEPs.

According to the trend of physiological indicators of two alfalfa varieties, it’s apparent to find that both of alfalfa varieties had notable changes under 35 and 43°C compared with 20°C. Therefore, the proteomic information of two alfalfa varieties exposed to 20, 35, and 43°C were analyzed by using TMT-LC/MS-MS. In this experiment, a total of 1,575,487 spectrum were generated through the TMA analysis. After searching the database of protein, the number of match spectrum was 184,025, which shows the utilization ratio of spectrum was 11.7%. A total of 93,785 peptides were identified at 95% confidence level through spectral analysis, in which has 88,958 unique peptides. A total of 7,300 proteins were identified, which has 6,704 quantifiable proteins (Supplementary Table 3). In order to analyze the differentially expressed proteins (DEPs) of two alfalfa varieties under HT stress, we compared the DEPs of MS30 and MS37 under different and same temperature respectively (Figure 3 and Supplementary Table 4). For MS30 and MS37, the number of DEPs were the most in the 43/35°C comparison group, with 1,235 and 1,355 respectively (Figures 3A,B). Under the same temperature comparison, the number of DEPs is the largest in the MS3035/MS3735 comparison group, which has a total of 575 DEPs and the up-regulated DEPs accounted for 71.3% (Figure 3C).

### Information-Identification and Trend Analysis of Differentially Expressed Proteins

#### Gene Ontology Analyses of Differentially Expressed Proteins in Response to High Temperature

**FIGURE 4:**
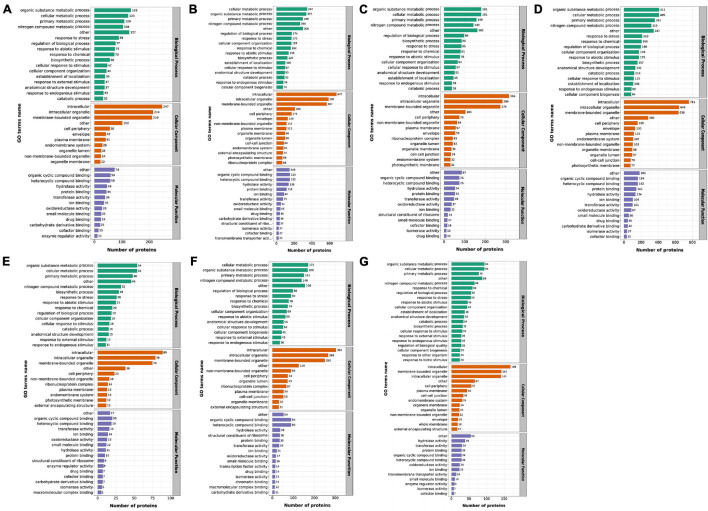
The functional distribution of DEPs in response to HT stress by GO level 2. The *x*-axis represents the number of enriched DEPs by GO annotation and the note number mean protein number. **(A)** The DEPs of MS3035/MS3020 by GO. **(B)** The DEPs of MS3043/MS3035 by GO. **(C)** The DEPs of MS3735/MS3720 by GO. **(D)** The DEPs of MS3743/MS3735 by GO. **(E)** The DEPs of MS3020/MS3720 by GO. **(F)** The DEPs of MS3035/MS3735 by GO. **(G)** The DEPs of MS3043/MS3743 by GO.

The GO database^1^ was used to annotate the DEPs. The result indicated that all identified DEPs were classified to different biological processes (BP) in which the major categories were response to organic substance metabolic process, cellular metabolic process, primary metabolic process and nitrogen compound metabolic process (Figures 4A–G). Among cellular components (CC), intracellular, intracellular organelle and membrane-bounded organelle were the most abundant groups. Additionally, in terms of molecular functions (MF), most of the DEPs were classified to the following categories: organic cyclic compound binding, heterocyclic compound binding, hydrolase activity and protein binding. In order to find the more specific GO items that play an important role under heat stress, the DEPs enrichment of the BP, CC and MF were analyzed in the GO classification, respectively (Supplementary Table 5). According to the results of the DEPs in the GO classification, we found that for MS30 and MS37, GO terms related to energy metabolism and photosynthetic processes appeared in 35/20 and 43/35°C comparison groups, such as cellular response to blue light, chloroplast inner membrane and acetyl-CoA biosynthetic process. In addition, we also discovered the dynamic changes of the DEPs in GO enrichment. For MS30, the DEPs were significantly enriched to the GO terms related to the cell wall in the 35/20°C comparison group, while in the 43/35°C comparison group, the DEPs were significantly enriched to GO term associated with PS II repair and chaperone repair. These results indicate that the photosynthetic processes and the repair process play an important role in the resistance of alfalfa to HT stress, especially under the stress of 43°C. We further compared the GO terms of the MS30/MS37 comparison group under 20, 35, and 43°C, and found that the synthesis of flavonoids and the process of RNA modification may be also related to the heat tolerance of plants.

#### Kyoto Encyclopedia of Genes and Genomes Analyses of Differentially Expressed Proteins in Response to High Temperature

The result of KEGG analysis^2^ show that for the MS30, most up-regulated DEPs in the 35/20°C comparison group enriched in pathways of Steroid biosynthesis, Circadian rhythm-plant, Protein processing in endoplasmic reticulum and Plant-pathogen interaction, whereas proteins involved in Porphyrin and chlorophyll metabolism were down-regulated (Supplementary Table 6). Most of DEPs related to Protein processing in endoplasmic reticulum and Carotenoid biosynthesis were increased, while DEPs associated with Biosynthesis of secondary metabolites-other antibiotics, Photosynthesis-antenna proteins, Photosynthesis and Ribosome were decreased in the 43/35°C comparison group. For the material MS37, the DEPs of the 35/20°C comparison group involved in the pathways of Photosynthesis-antenna proteins, Linoleic acid metabolism, alpha-Linolenic acid metabolism and Photosynthesis were up-regulated, whereas DEPs in Zeatin biosynthesis were down-regulated. In the 43/35°C comparison group, the up-regulated DEPs were significantly enriched in pathways of Monobactam biosynthesis and Protein processing in endoplasmic reticulum, while down-regulated DEPs were significantly enriched in Photosynthesis-antenna proteins, Photosynthesis-antenna proteins and Linoleic acid metabolism. We further analyzed the KEGG enrichment of DEPs in the MS30/MS37 comparison group under 20, 35, 43°C, respectively, and also found pathways related to Glucosinolate biosynthesis, alpha-Linolenic acid metabolism and Diterpenoid biosynthesis. In addition, pathways such as Ribosome, Porphyrin and chlorophyll metabolism, Lysine biosynthesis and Thiamine metabolism also appeared in each comparison group of MS30/MS37. The dynamic changes of these pathways reflect the response mode of two alfalfa varieties under high temperature stress and are related to their heat tolerance.

### Key Proteins Among Differentially Expressed Proteins in Response to High Temperature Stress

**FIGURE 5:**
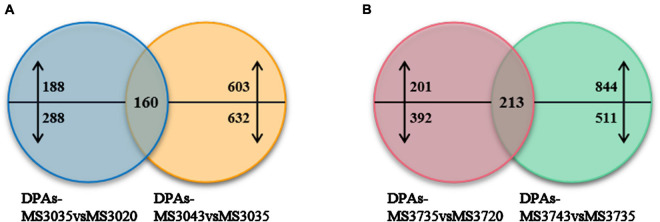
Venn diagrams of two alfalfa varieties. **(A)** Venn diagrams of MS30. **(B)** Venn diagrams of MS30. Venn diagrams show the number of common, significantly up-regulated and down-regulated proteins in two alfalfa varieties under high temperature stress, up- and downward arrows represent up-regulated and down-regulated proteins, respectively.

According to the results of GO and KEGG enrichment, we found that the DEPs were mainly enriched in the processes of photosynthesis, energy, damage and repair. Therefore, we constructed the Venn diagrams of MS30 and MS37 under HT stress, respectively (Figure 5). And the DEPs associated with photosynthesis, regulation, repair and metabolism were selected in the overlapping regions of the Venn diagrams to analyze their dynamic changes under HT stress.

#### Photosynthesis-Related Differentially Expressed Proteins

**FIGURE 6:**
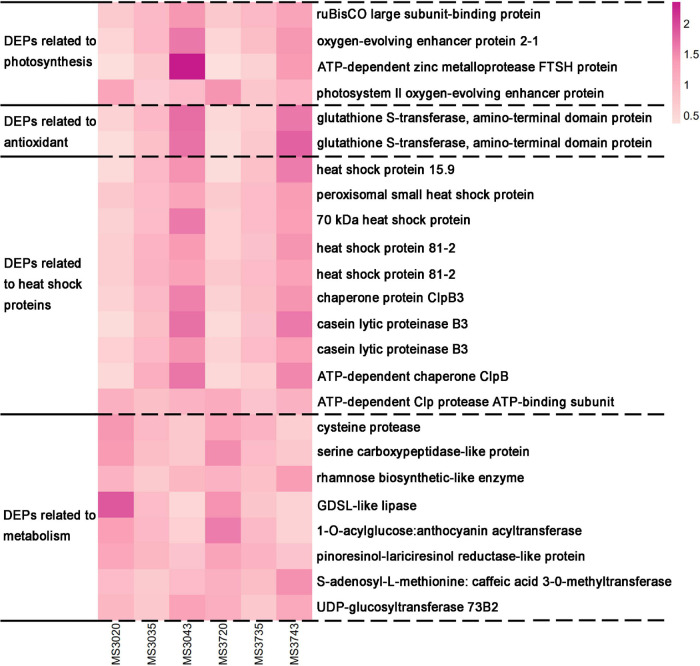
The heatmap of key DEPs co-existed by both alfalfa varieties in response to HT stress. Heat map based on the expression leave of each protein in each sample. The darker the color, the higher the protein expression, and the lighter the color, the lower the protein expression. Each row in the heat map represents the levels of a DEP under different conditions. The DEP name were listed on the right side of the heat map.

Venn diagram analysis was illustrated the numbers of common DEPs in both comparison groups of MS30 is 160 (Figure 5A). Among these 160 DEPs, a total of 10 DEPs are involved in the photosynthetic process under high temperature stress, including those related to photosynthetic system II, chlorophyll, calvin cycle and light photoreceptor (Supplementary Table 7A). Among these DEPs, there are four DEPs showing an up-regulated trend and two DEPs showing a down-regulated trend as the temperature rises. For the material MS37, the Venn diagram analysis was illustrated that 213 DEPs were located in the repeated regions of the two comparison groups (Figure 5B). Among them, there are a total of 15 DEPs related to photosynthesis (Supplementary Table 7B). We observed that four DEPs related to photosynthesis co-exist in MS30 and MS37, and their accumulation trends in these two varieties were consistent, which indicates that these DEPs play an important role in alfalfa response to HT stress (Figure 6). It is noteworthy that among these four DEPs, three DEPs have been always up-regulated under the HT stress, and their increased accumulation in MS30 was significantly higher than that in MS37. Considering the difference in heat resistance of two varieties, we infer that these three DEPs could be one of the key factors that determine the heat resistance of alfalfa.

#### Antioxidant-Related Differentially Expressed Proteins

Here, we observed that HT stress altered the abundance of five DAPs in the both comparable groups of MS30, and six DAPs in the both comparable groups of MS37, which is mainly related to glutathione (Supplementary Tables 8A,B). Among these DEPs, two DEPs related to glutathione S-transferase co-exist in MS30 and MS37, and have always been up-regulated under the HT stress (Figure 6). In addition, we also found two other DEPs related to glutathione S-transferase in MS37, and they were also up-regulated during the whole process of high temperature stress. These results indicate that glutathione S-transferase could play a positive role in alfalfa response to HT stress.

#### Different Expressed Heat Shock Proteins

The number of different expressed heat shock proteins (DEHSPs) that were found in both comparison groups of MS30 is thirty-six, which is more than the number of DEHSPs that were found in both comparison groups of MS37 (Supplementary Tables 9A,B). In additional, we found the DEHSPs in MS30 includes all classes of heat shock proteins, but the DEHSPs in MS37 only includes five classes of heat shock proteins, sHSP, HSP70, HSP90, HSP100, and FKBP. Comparing the accumulation trends of the DEHSPs shared by MS30 and MS37, we found that almost all DEHSPs were up-regulated with increasing temperature, only ATP-dependent Clp protease ATP-binding subunit (TRINITY_DN17376_c2_g3_Gene_31004) has been changed in a fluctuating way, which is down-regulated in the 35/20°C comparable group and up-regulated in the 43/35°C comparable group (Figure 6). Moreover, the accumulations of the DEHSPs that has always been up-regulated in MS30 are higher than that in MS37 at 35°C. These results indicate that under the HT stress, the difference in HSPs between the two varieties largely affects their heat resistance.

#### Metabolism-Related Differentially Expressed Proteins

**FIGURE 7:**
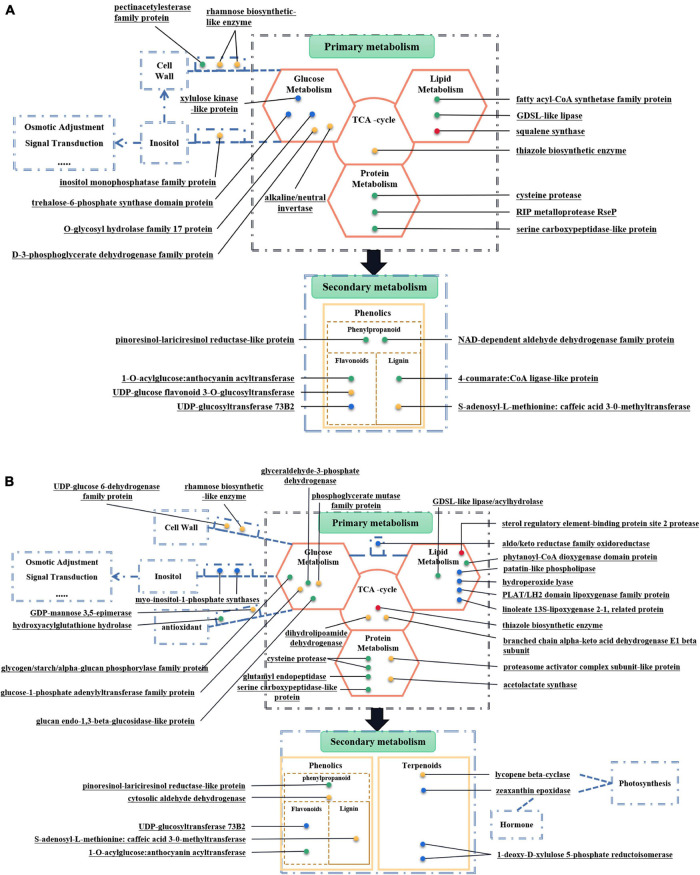
Metabolism-Related DEPs of two alfalfa varieties. **(A)** Metabolism-Related DEPs in MS30. **(B)** Metabolism-Related DEPs in MS37. These figures can be divided into two parts, including primary metabolism and secondary metabolism. Each dot represents a protein, which plays a role in the corresponding metabolic process. The green dots indicate proteins that have been down-regulated throughout the process of HT stress. The red dots indicate proteins that have been up-regulated throughout the process of HT stress. The blue dots indicate the protein whose expression level rises first and then falls during the entire process of HT stress. The yellow dots indicate proteins whose expression levels first decrease and then increase during the entire process of high temperature stress.

There are twenty-three metabolism-related DEPs in both comparison groups of MS30 (Supplementary Table 10A). Among these DEPs, sixteen DEPs are related to primary metabolism and seven DEPs are related to secondary metabolism (Figure 7A). In both comparison groups of MS37, we found thirty-seven metabolism-related DEPs, including twenty-eight DEPs related to primary metabolism and nine DEPs related to secondary metabolism (Figure 7B and Supplementary Table 10B). Analyzing the DEPs shared by MS30 and MS37, we found that under the HT stress, the accumulation trends of these DEPs in the two alfalfa varieties are exactly the same (Figure 6). All these results indicated that HT stress had an effect on the metabolism of the two alfalfa varieties, but from the number of metabolism-related DEPs and the metabolic pathways involved in these DEPs, the HT stress has a greater impact on the MS37.

### Validation of Candidate Functional Protein Selections by Parallel Reaction Monitoring

Among the DEPs in MS30 and MS37, those having predicted annotations related to “photosynthesis,” “regulation,” “repair,” and “metabolism” are most likely to be responsive to HT stress. Therefore, we annotated the DEPs that shared by the two alfalfa varieties, MS30 and MS37. In order to validate the TMT results, 14 DEPs were randomly selected for targeted parallel reaction monitoring (PRM) assay, of which 11 proteins had quantitative information and showed a good consistency with TMT results, including one DEP related to oxidation-reduction, two DEPs related to photosynthesis, four DEPs related to heat shock proteins and four DEPs related to metabolism in both MS30 and MS37 (Table 1).

**TABLE 1:** Comparison between the isobaric tandem mass tag labeling (TMT) for relative quantitation and parallel reaction monitoring (PRM).

Pathway	Protein Accession	Protein description	MS3035/MS3020 Ratio	MS3043/MS3035 Ratio	MS3035/MS3020 Ratio (TMT)	MS3043/MS3035 Ratio (TMT)	MS3735/MS3720 Ratio	MS3743/MS3735 Ratio	MS3735/MS3720 Ratio (TMT)	MS3743/MS3735 Ratio (TMT)
Photosynthesis	TRINITY_DN12545_ c0_g1_Gene_9459	magnesium-chelatase subunit ChlI	0.15	4.29	0.56	1.31	0.22	2.63	0.52	1.38
	TRINITY_DN16844_ c0_g3_Gene_8166	oxygen-evolving enhancer protein 2-1	3.07	2.54	1.82	1.68	3.30	2.11	1.68	1.59
Antioxidant activities	TRINITY_DN18935_ c0_g3_Gene_24344	glutathione S-transferase, amino-terminal domain protein	3.69	2.53	2.11	1.95	2.28	3.08	1.67	2.44
Heat shock proteins	TRINITY_DN10929_c0 _g1_Gene_4083	heat shock protein 81-2	2.97	1.85	1.65	1.34	1.88	2.71	1.36	1.69
	TRINITY_DN21025_ c0_g2_Gene_8907	70 kDa heat shock protein	1.57	2.08	1.56	1.77	1.56	2.42	1.47	1.48
	TRINITY_DN17376_ c2_g3_Gene_31004	ATP-dependent Clp protease ATP-binding subunit	0.65	2.39	0.79	1.22	0.63	2.08	0.67	1.36
	TRINITY_DN14499_ c0_g1_Gene_14445	peroxisomal small heat shock protein	1.44	2.38	1.27	1.34	1.90	2.11	1.27	1.49
Metabolism	TRINITY_DN14955 _c2_g1_Gene_12704	cysteine protease	0.53	0.78	0.67	0.79	0.70	0.52	0.83	0.63
	TRINITY_DN14760_c0 _g2_Gene_11432	rhamnose biosynthetic-like enzyme	0.45	3.28	0.68	1.40	0.66	3.07	0.80	1.60
	TRINITY_DN22711_c0 _g1_Gene_33078	1-O-acylglucose: anthocyanin acyltransferase	0.55	0.52	0.72	0.63	0.38	0.60	0.59	0.61
	TRINITY_DN10050_c0 _g2_Gene_32110	S-adenosyl-L-methionine: caffeic acid 3-0-methyltransferase	0.50	2.20	0.78	1.30	0.64	2.79	0.83	1.63

## Discussion

### The Change of Photosynthesis Under Heat Stress

Photosynthesis is a process by which plants use light energy to assimilate carbon dioxide (CO_2_) and water (H_2_O) to produce organic substances and release oxygen, which is closely related to plant growth and productivity (Yamamoto, 2001; Foyer et al., 2017). However, when plants are exposed to heat stress, photosynthesis is the most sensitive physiological process (Wahid et al., 2007). In this study, we exposed alfalfa plants to periodically increasing high temperature treatment as heat stress. Our data showed that the photosynthetic system of both alfalfa materials was affected, which was reflected in physiological level and protein level. In terms of physiological changes, although the Fv/Fm shows a downward trend in both materials, the decrease of Fv/Fm in MS37 is significantly stronger than that in MS30. And the chlorophyll content of MS37 also showed a decrease trend with the increase of temperature, but that of MS30 showed fluctuating changes. These results revealed that the photosynthesis of the MS37 was significantly stronger affected than that of MS30 under the heat stress. Similar conclusions have been reported in the study of heat resistance of wheat (Sharma et al., 2015).

In terms of protein expression, our data found that heat stress altered the abundance of ten proteins in MS30 and fifteen proteins in MS37 related to photosynthesis. Of these, there are four DEPs co-existing in MS30 and MS37, which is RuBisCO large subunit-binding protein (TRINITY_DN17135_c0_g1_Gene_34667), oxygen-evolving enhancer protein 2-1 (TRINITY_DN16844_c0_g3_Gene_8166), ATP-dependent zinc metalloprotease FTSH protein (TRI NITY_DN21254_c0_g1_Gene_19039) and magnesium-chelatase subunit ChlI (TRINITY_DN12545_c0_g1_Gene_9459). The RuBisCO large subunit-binding protein has two different types of subunit, termed alpha and beta, and some studies suggest this protein is implicated in the assembly of Rubisco in higher plant chloroplasts (Musgrove and Ellis, 1986). Rubisco plays an important role in plant growth, which catalyzes the photosynthetic assimilation of CO_2_ into organic compounds (Carmo-Silva et al., 2015). In this experiment, the RuBisCO large subunit-binding protein has been up-regulated in the two alfalfa materials, which could contribute to enhance heat tolerance of alfalfa. Oxygen-evolving enhancer protein 2-1 reported in Arabidopsis thaliana and *Nicotiana tabacum* may be involved in the regulation of photosystem II^3^. More specifically, the function of oxygen-evolving enhancer protein is related to photosynthetic oxygen evolution and it is thought to optimize the manganese cluster during water oxidation (Heide et al., 2004). Moreover, the oxygen evolution enhancer protein could respond to heat stress and protects the reaction center D1 protein (Yamamoto, 2001). Therefore, this protein was up-regulated in two alfalfa materials could contribute to enhance heat tolerance of alfalfa. ATP-dependent zinc metalloprotease FTSH protein has been reported to play an important role in plant heat resistance (Chen et al., 2006). That is because FtsH plays a critical role in the biogenesis of thylakoid membranes, quality control in the photosystem II repair cycle and photosynthetic electron-transport pathways (Kato and Sakamoto, 2018). In this experiment, the expression and up-regulation trend of this protein in MS30 were significantly higher than that of MS37, especially under the HT stress of 43°C. And it is noteworthy that in the MS30 we found another ATP-dependent zinc metalloprotease FTSH protein (TRINITY_DN21254_c0_g1_Gene_19039) has also been up-regulated under the HT stress of 43°C. Therefore, this DEP may be one of the factors for the difference in heat resistance between the two alfalfa varieties.

In MS37, we find the DEPs related to light-harvesting complex I and photosystem I reaction center, which has been up-regulated under the HT stress of 35°C and down-regulated under 43°C. This result indicates that the MS37 has a certain heat resistance ability under the high temperature stress of 35°C, but under 43°C, the heat resistance ability is obviously reduced, and the structure and function of photosystem I are destroyed. In addition, photosystem II reaction center PsbP family protein (TRINITY_DN21665_c0_g1_Gene_23571) had always been down-regulated during heat stress. PsbP protein could stabilize and coordinate supramolecular organization and function of PSII, which plays an important role in the dynamic life cycle of PSII (Ifuku et al., 2011). And the expression of PsbP protein is beneficial to the oxygen-evolving activity and photoautotrophic growth of plants (Nishimura et al., 2017). The other DEP related PSII is vitamin K epoxide reductase family protein (TRINITY_DN17556_c0_g3_Gene_31603) in MS37. Studies have shown that the deletion of vitamin K epoxide reductase (VKOR) gene will reduce the photosynthetic efficiency of plants and affect the stability of PSII assembly (Du, 2015). In this experiment, VKOR protein was up-regulated under the heat stress of 35°C, but down-regulated under 43°C, indicating that the protein could respond to a certain intensity of heat stress. At the same time, we infer that the Change in expression of photosystem II reaction center PsbP family protein (TRINITY_DN21665_c0_g1_Gene_23571) and vitamin K epoxide reductase family protein (TRINITY_ DN17556_c0_g3_Gene_31603) may be associated with the significant decrease of Fv/Fm in MS37. Taken together, the above results indicated that the effect made by HT stress to Photosynthesis in MS37 is stronger than that in MS30. And the physiological and proteomic data indicate that MS37 has a certain degree of heat resistance under 35°C heat stress, but MS37 is severely affected by 43°C heat stress, which explains the difference in heat resistance of the two varieties.

### The Change of Substance Metabolism Under High Temperature Stress

It has been reported that heat stress, drought stress, salt stress, etc., can cause damage to plant growth, and at the same time induce the production of substances related to anti-oxidation and osmotic adjustment to maintain the homeostasis in plants (He et al., 2012; Rivero et al., 2014; Soltabayeva et al., 2021). In this experiment, the results of MDA and EL indicate under HT stress, the membrane systems of the two alfalfa varieties were damaged, especially under 43°C HT stress. And the membrane system of MS37 is more severely damaged than that of MS30. In terms of osmotic regulators substances, induced by HT stress, the contents of proline, soluble protein and soluble sugar of the two alfalfa varieties have all changed. But on the whole, the osmotic adjustment substance of MS30 is significantly more responsive to HT stress than that of MS37. In terms of antioxidant systems, we get the same result that the activity of antioxidants in both alfalfa varieties were affected by HT stress. Therefore, the physiological results indicate heat stress affected the substance content and activity in both alfalfa varieties, which involves changes of the substances metabolism in alfalfa and is related to the heat resistance of alfalfa.

#### The Change of Primary Metabolism Under High Temperature Stress

We found that HT stress respectively altered the abundance of twenty-three and thirty-eight proteins related to metabolism in MS30 and MS37. There are sixteen DEPs related to primary metabolism in MS30, of which DEPs related to carbohydrate metabolism are the most numerous. It is noteworthy that we found that carbohydrate metabolism involves the metabolism of inositol. Inositol has important biological functions in plants, which not only participates in the formation of cell walls and cell membranes, but also plays an important role in various life activities such as osmotic regulation, signal transduction, and stress regulation (Zhang et al., 2013; Johnen, 2021). Inositol monophosphatase (IMPase) is a catalytic enzyme for the final step of inositol biosynthesis (Goswami et al., 2018). Studies have shown that abiotic stresses such as low temperature, drought and high salinity can affect the activity of IMPase (Nourbakhsh et al., 2015). In this experiment, inositol monophosphatase family protein (TRINITY_DN9184_c0_g3_Gene_30850) is up-regulated under 43°C HT stress, indicating that MS30 could promote the inositol synthesis through carbohydrate metabolism pathway, which could contribute to enhance heat tolerance of MS30. We have also found DEPs, myo-inositol 1-phosphate synthase (TRINITY_DN18700_c1_g2_Gene_12271) and myo-inositol-1-phosphate synthases (TRINITY_DN21940_c0_g2_Gene_13532), associated with inositol synthesis in MS37. Myo-inositol 1-phosphate synthase (MIPS) is a key enzyme that catalyzes D-Glucose-6-phosphate to form Myo-inositol-1-phosphate (Yang et al., 2017). Studies have shown that MIPS genes from different species play an important role in plant stress resistance (Majee et al., 2004; Das-Chatterjee et al., 2006; Guo et al., 2017). In this experiment, the DEPs related to inositol synthesis in MS37 are up-regulated under 35°C HT stress, but down-regulated under 43°C, indicating that MIPS contribute to the heat resistance of alfalfa under a certain degree of heat stress. Therefore, the results of MS30 and MS37 show that HT stress can cause changes in inositol metabolism in alfalfa, thereby affecting the heat resistance of alfalfa. In addition, among DEPs related to carbohydrate metabolism, we also found DEPs related to plant cell walls. Rhamnose is an important component in the cell wall and participates in many metabolic processes in plants (Liu et al., 2019; Jiang et al., 2020). Rhamnose synthase catalyze the initial step of the synthesis of rhamnose (Han, 2014). In this experiment, we found that rhamnose biosynthetic-like enzyme (TRINITY_DN14760_c0_g2_Gene_11432) coexisted in two alfalfa varieties, both were down-regulated at 35°C high temperature stress, and up-regulated at 43°C. Moreover, there is another rhamnose biosynthetic-like enzyme (TRINITY_DN17882_c0_g1_Gene_10641) showed the same change trend in MS30, which indicates that high temperature stress may affect the structure and function of alfalfa cell wall through rhamnose metabolism pathway.

In this experiment, we also found that high temperature stress affected the TCA cycle of MS30 and MS37. Thiazole biosynthetic enzyme is not only related to plants growth and development, but also plays an important role in stress resistance. This is because thiazole biosynthetic enzyme is involved in plant thiamine biosynthesis (Sun et al., 2018). Plant thiamine could be response to oxidative stress by scavenging free radical and involve in TCA cycle, pyruvate carboxylase, pyruvate oxidase as an essential coenzyme (Zhou et al., 2012; Sun et al., 2018). In this experiment, thiazole biosynthetic enzyme as a DEP co-existed in both alfalfa varieties. In MS30, thiazole biosynthetic enzyme (TRINITY_DN20649_c0_g2_Gene_9792) was up-regulated at 43°C, and its expression was significantly higher than that at 20°C. In MS37, thiazole biosynthetic enzyme (TRINITY_DN11811_c1_g1_Gene_35054) has been always up-regulated under high temperature stress. These results indicate that high temperature stress can affect the TCA cycle and heat resistance of alfalfa by affecting the expression of thiazole biosynthetic enzyme.

#### The Change of Secondary Metabolism Under High Temperature Stress

Plant secondary metabolites play important roles for plants to improve defensive and competitive abilities as well as correspond to the environments (Yan et al., 2007). In this experiment, our data found that HT stress altered the abundance of seven and nine proteins related to secondary metabolism in MS30 and MS37, respectively. In MS30, the seven DEPs are all involved in the biosynthesis of phenolic compounds in plant secondary metabolites. But in MS37, except for five DEPs involved in the biosynthesis of phenolic compounds, there are four DEPs involved in the biosynthesis of terpenoids. In the biosynthesis of phenolic compounds, there are four DEPs coexist in both alfalfa varieties. Of these, 1-O-acylglucose: anthocyanin acyltransferase (TRINITY_DN22711_c0_g1_Gene_33078) and pinoresinol-lariciresinol reductase-like protein (TRINITY_ DN16684_c0_g2_Gene_19375) have always been down-regulated under the high temperature stress. 1-O-acylglucose: anthocyanin acyltransferase has the function of acyltransferase, which can catalyze the conversion of glycosylated anthocyanins into acylated anthocyanins. Studies have shown that acylated anthocyanins have higher thermal stability and can enhance the antioxidant properties of purple cabbage (Yuan, 2018). Pinoresinol-lariciresinol reductase (PLR) are enzymes involved in the lignan biosynthesis after the initial dimerization of two monolignols (Xiao et al., 2021). Some studies have also shown lignans play a role in defense against oxidative stress (Li et al., 2006; Markulin et al., 2019). In this experiment, the accumulations of these two proteins were down-regulated under the HT stress, indicating that the metabolic pathways of anthocyanin and lignan were affected. Therefore, we speculate that HT stress may cause damage to alfalfa by reducing the effects of anthocyanins and lignan in plants. In addition, the enzyme involved in the metabolism of lignin, S-adenosyl-L-methionine: caffeic acid 3-0-methyltransferase (TRINITY_DN10050_c0_g2_Gene_32110), has been down-regulated at 35°C and up-regulated at 43°C in both alfalfa varieties. S-adenosyl-L-methionine: caffeic acid 3-0-methyltransferase (COMT) is involved in S lignin biosynthesis. It is worth noting that 4-coumarate: CoA ligase-like protein (TRINITY_DN17517_c0_g1_Gene_20937) in MS30 has been down-regulated under HT stress. 4-coumarate: coenzyme A ligase (4CL) converts 4-coumaric acid and its hydroxylated derivatives into the CoA thiol esters, directing carbon flux into various end-products of phenylpropanoid metabolism, such as flavonoids and lignins (Wohl and Petersen, 2020). Studies have shown that inhibiting the activity of COMT and 4CL could lead to a decrease in lignin content (Awasthi et al., 2019; Wohl and Petersen, 2020). Therefore, we speculate that HT stress could also affect the heat tolerance of alfalfa by affecting the metabolic pathway of lignin.

Terpenoids are the most abundant and diverse compounds in plant secondary metabolites (Caputi and Aprea, 2011). In this experiment, HT stress affected the metabolic of terpenoids in MS37. Among the DEPs related to the biosynthesis of terpenoids in MS37, lycopene beta-cyclase (TRINITY_DN16506_c0_g1_Gene_6592) has been down-regulated at 35°C and up-regulated at 43°C. Lycopene beta-cyclase (LCYB) participates in the metabolic pathway of carotenoids (Yamaguchi, 2012). Lots of studies have shown that high expression of LCYB gene can significantly increase carotenoid biosynthesis and enhance the tolerance of plants to abiotic stress (Jia et al., 2011; Chen et al., 2018; Li Y. et al., 2018). In this experiment, HT stress affected the expression of LCYB, indicating that HT stress could affect the photosynthesis and heat tolerance of alfalfa through the metabolic pathway of carotenoids. The other DEPs related to the metabolic of terpenoids is 1-deoxy-D-xylulose 5-phosphate reductoisomerase (TRINITY_DN13041_c3_g1_Gene_16656) and 1-deoxy-D-xylulose 5-phosphate reductoisomerase (TRINITY_DN13041_c3_g1_Gene_16657). 1-deoxy-D-xylulose 5-phosphate reductoisomerase (DXR) catalyzes the second step of the methylerythritol phosphate (MEP) pathway that functions in several organisms and plants for the synthesis of isoprenoids (Takahashi et al., 1998; Kesharwani and Sundriyal, 2020). Studies have shown that inhibiting or increasing the DXR expression will affect the production of terpenes, thereby affecting photosynthesis (Hasunuma et al., 2008; Wang et al., 2018). Therefore, combined with MS37 photosynthesis results, we speculate that a certain degree of HT stress will promote the synthesis of terpenoids in MS37, so as to maintain the photosynthesis of MS37. But excessive temperature will also inhibit the synthesis of terpenoids, which may be one of the factors leading to the decrease of the Fv/Fm ratio and the chlorophyll content in MS37 under 43°C HT stress.

### The Change of Heat Shock Proteins Under Heat Stress

Heat shock proteins are molecular chaperones fulfiling stress-related and housekeeping functions, including protein folding, assembly, degradation and translocation, thus playing a central role in protein homeostasis (Fragkostefanakis et al., 2015). Here, we observed thirty-six and thirteen HSPs in MS30 and MS37 were differentially accumulated in response to heat stress, respectively. These DEHSPs were almost up-regulated during the entire high temperature stress, which indicated HSPs in both alfalfa varieties were playing an active role to protect alfalfa from high temperature stress. But it is noteworthy that compared with MS37, MS30 has more HSP quantity and classes. These results indicate that the difference in DEHSPs could be one of the reasons why MS30 has better heat tolerance than MS37.

When plants are subjected to abiotic stress, a large number of misfolded or unfolded proteins will accumulate in the endoplasmic reticulum, which can trigger endoplasmic reticulum stress (ERS). Studies have shown that ERS can induce an increase in the expression of various molecular chaperones and their auxiliary partners, such as HSPs, because they play important functions in Endoplasmic reticulum quality control (ERQC), unfolded protein response (UPR), and ER-associated degradation (ERAD) (Zhao et al., 2007; Fu et al., 2016). In this experiment, we found thirteen and five DEHSPs related to Protein processing in endoplasmic reticulum (KEEG: mtr04141) in MS30 and MS37, respectively. Among these, four DEHSPs (TRINITY_DN11338_c0_g1_Gene_18098, TRINITY_ DN21025_c0_g2_Gene_8907, TRINITY_DN10929_c0_g1_Gene _4083, TRINITY_DN17416_c0_g2_Gene_3822) co-exist in two alfalfa varieties and have always been up-regulated under the HT stress, which indicates that these four DEHSPs can protect alfalfa from high temperature stress by regulating the endoplasmic reticulum stress. In addition, compared with MS37, MS30 has more DEHSPs involved in Protein processing in endoplasmic reticulum under HT stress, which suggests that MS30 can more actively respond to ERS to maintain the homeostasis and function of the endoplasmic reticulum. We also found five HSP60s were up-regulated in MS30 under the HT stress, which is absent in MS37. It has been reported that overexpression of HSP60 could improve plant stress resistance, while inhibiting the expression of HSP60 could affect the normal growth and development of plants (Hill and Hemmingsen, 2001; Cabiscol et al., 2002). These results clarify the reasons for the strong heat resistance of MS30. HSP100/ClpB (casein lytic proteinase B) could function in a non-degradative pathway of protein renaturation to liberate native functional proteins from aggregates, which is important to reactivate heat aggregated proteins under demanding, stressful conditions (Mishra and Grover, 2016). In this experiment, our data found that HT stress altered respectively the abundance of seven and six ClpB proteins in MS30 and MS37. Of these, we found that three ClpB3 proteins shared by two alfalfa varieties were up-regulated under HT stress. ClpB3 specifically targets unstructured polypeptides and mediates the reactivation of heat-denatured model substrates in an Hsp70-independent manner (Parcerisa et al., 2020). Therefore, we speculate that ClpB3 proteins play an important role in the response of alfalfa to HT stress, and ClpB3 proteins were up-regulated under HT stress could contribute to enhance heat tolerance of alfalfa.

## Conclusion

**FIGURE 8:**
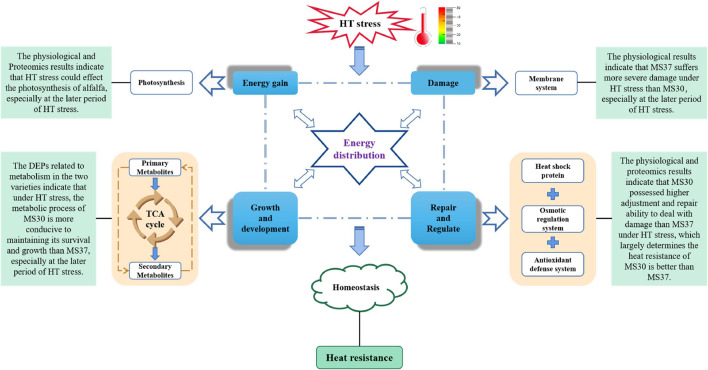
Model shows the differential responses of two alfalfa varieties with contrasting heat tolerance to HT stress based on physiological and proteomic changes.

In summary, a model of HT-mediated stress response in *Medicago sativa* L. was proposed by conducting a comprehensive comparative analysis of two alfalfa varieties contrasting in heat tolerance based on their physiological and proteomic changes (Figure 8). HT induces the ROS accumulation and osmotic pressure in alfalfa, which significantly weakens the photosynthetic efficiency, destroys the cell membrane structure and causes endoplasmic reticulum stress. Our results demonstrated that MS30 was more tolerant to HT stress than MS37, which was evidenced by the differences at the physiological and proteomic levels. MS30 possessed higher abilities of adjustment and repair to deal with damage than those of MS37 under HT stress conditions. Meanwhile, the proteomic analysis revealed the metabolic process of MS30 is more conducive to maintaining its survival and growth than MS37, especially at the later period of HT stress. All these processes involve energy distribution and the maintenance of homeostasis in alfalfa, which ultimately determines the heat tolerance of alfalfa. These results provide basic information for exploring the physiological and molecular mechanisms of alfalfa adaptation or tolerance to HT stress. Nevertheless, a challenge for future work will be to elucidate the complex regulation networks of HT response and to identify key regulators and their function in the context of high temperature, which could contribute to accelerate the breeding progress of high-quality alfalfa varieties with heat resistance.

## Data Availability Statement

The datasets presented in this study can be found in online repositories. The names of the repository/repositories and accession number(s) can be found in the article/Supplementary Material.

## Author Contributions

YL, XLi, and JY performed the experiments. YL, XLi, and JinZ analyzed the data and prepared the figures and tables. JY, XLi, and JinZ edited and revised the manuscript. XLi, JinZ, DL, LY, MY, JiaZ, DC, XJ, XLe, JA, ML, and SB conceived, designed research, and wrote this manuscript. YL, XLi, JinZ, DL, LY, MY, JiaZ, DC, XJ, XLe, JA, ML, SB, and JY approved the final version of this manuscript. All authors contributed to the article and approved the submitted version.

## Conflict of Interest

The authors declare that the research was conducted in the absence of any commercial or financial relationships that could be construed as a potential conflict of interest.

## Publisher’s Note

All claims expressed in this article are solely those of the authors and do not necessarily represent those of their affiliated organizations, or those of the publisher, the editors and the reviewers. Any product that may be evaluated in this article, or claim that may be made by its manufacturer, is not guaranteed or endorsed by the publisher.

## Footnotes

1
http://www.ebi.ac.uk/GOA/

2
http://www.genome.jp/kegg/

3
https://www.uniprot.org/

## Abbreviations

2-DE: two-dimensional gel electrophoresis
APX: ascorbate peroxidase
CAT: catalase
EL: electrolyte leakage
DEPs: differentially expressed proteins
Fv: variable fluorescence
Fm: maximal fluorescence
Fv/Fm: the chlorophyll fluorescence parameter
GO: gene ontology
GSH-Px: glutathione peroxidase
H2O2: hydrogen peroxide
Hsps: heat shock proteins
KEGG: Kyoto Encyclopedia of Genes and Genomes
LC-MS/MS: liquid chromatography-tandem mass spectrometry
MDA: malondialdehyde
POD: peroxidase
PRM: parallel reaction monitoring
Pro: Proline
ROS: reactive oxygen species
RuBisCO: Ribulose-1,5-bisphosphate carboxylase/oxygenase
SOD: superoxide dismutase
SSC: soluble sugar content
TMT: tandem mass tags.
